# Embodied planning in climbing: how pre-planning informs motor execution

**DOI:** 10.3389/fpsyg.2024.1337878

**Published:** 2024-02-19

**Authors:** Vicente Luis-del Campo, Jesús Morenas Martín, Lisa Musculus, Markus Raab

**Affiliations:** ^1^Faculty of Sports Sciences, University of Extremadura, Cáceres, Spain; ^2^Institute of Psychology, Department of Performance Psychology, German Sport University Cologne, Cologne, North Rhine-Westphalia, Germany

**Keywords:** embodied cognition, expertise, eye-tracking, visual behavior, movement, climbing

## Abstract

**Introduction:**

The aim of the study is to address embodied planning in climbing. Embodied planning was conceptualized as the interaction between perceptual-cognitive pre-planning and motor execution.

**Methods:**

In an experimental study, 18 climbers were asked to pre-plan a climbing route and to perform the route afterward. During pre-planning, the visual search pattern of climbers was captured using a portable eye tracker. After previewing, they were invited to climb the wall.

**Results:**

Results revealed that holds looked at during pre-planning were used twice as much during route execution than those not looked at. The duration of fixations was longer for holds used than those not used during route execution. The experience of climbers (training years) correlated with visual strategies and climbing performance, such that experienced participants climbed faster and fixated at the holds not used for a shorter time.

**Discussion:**

The visual behaviors of climbers were influenced by their past sensorimotor experiences during route previewing, impacting subsequent climbing performance.

## Introduction

Climbing was introduced as a new sport discipline in the Olympic Games in Tokyo in 2020 due to its popularity and professionalization around the world (Künzell et al., 2021). The International Federation of Sport Climbing (IFSC, 2023) differentiates three types of indoor climbing disciplines. Specifically, these are known as ‘Lead’ (athletes climb secured by a rope, one at a time, on an overhanging route with a 6-min time limit; the aim is to go as high as possible in an individual attempt on a 15 m wall); ‘Speed’ (secured from above, climbers run up standardized parallel routes; the aim is to be the fastest to reach the top of a 15 m wall); and ‘Boulder’ (these competitions take place on 4-meter-high walls equipped with safety mats; the aim is to solve the highest number of problems – i.e., routes – on four/five round-dependent boulders, having an initial collective observation time of 2 min without yet attempting to complete the routes). There are also three climbing styles to successfully climb to the top of a route: ‘On-sight,’ where the climber performs a route without prior knowledge of it; ‘Flash,’ where the climber completes a climb on the first attempt after receiving guidance on the route; and ‘Redpoint,’ where the climber completes a route without falling after several unsuccessful attempts, rappelling down the route or interceding with a top rope.

A crucial factor for climbing performance is the development of an efficient climbing style, which entails interactions between the perceptual, cognitive, and motor system (Saul et al., 2019). Recently, the theoretical concept of embodied planning has been put forward, and specific predictions about the motor-cognitive interactions underlying climbing performance have been formulated (Musculus et al., 2021). The present study aims to test embodied planning predictions by particularly focusing on the impact of pre-planning on subsequent motor execution for the ‘On-sight’ style in indoor climbing (e.g., a climb without previous practice).

In climbing, climbers engage in route previewing before approaching the wall and executing the route. During this phase, climbers take a close look at the layout of the holds and plan which holds to use. After this initial previewing, climbers approach the wall and start climbing the route. In climbing research, experimental studies have examined the effects of route previewing in the initial pre-planning phase on subsequent climbing performance. Previewing is related to successful climbing performance (Sanchez et al., 2012, 2019; Seifert et al., 2017). Climbing studies focusing on pre-planning have used perceptual measures such as visual search behavior (Seifert et al., 2017; van Knobelsdorff et al., 2020), estimations of hold properties (e.g., Bläsing et al., 2014), and estimations of whether holds could be reached or not (e.g., Seifert et al., 2021a). These perceptual measures are then related to the number of holds used or the time needed to execute the route (Nieuwenhuys et al., 2008; Sanchez et al., 2012; Seifert et al., 2017).

From an embodied cognition perspective, the initial or pre-planning during the previewing of a climbing route depends on both the spatial information of specific hold positions and the climbers’ action capabilities. This means that the perception of the spatial properties of the environment is scaled to the ability of each performer who will execute the intended movements (Paterson et al., 2013). From this embodied perspective, the climber should not only perceive the spatial location of each hold at the climbing wall but also associate specific opportunities for action based on the sequence of holds available in the specific environment. Therefore, in this study we aim to directly capture the potential effects of the environment by analyzing climbing routes in a fine-grained manner, i.e., by inspecting the functional relation between gaze and movement execution on the level of holds. Furthermore, it has been suggested that climbing expertise produces action capabilities at different skill levels, such that advanced climbers showed greater maximal reaching distances than intermediate climbers. However, no effect of climbing expertise on their calibration accuracy was found because the different expertise groups estimated their maximal reaching distance similarly when this estimation was scaled to their actual maximal reaching distance (Seifert et al., 2021b). Therefore, climbers of all skill levels need to consider their bodily state as well as physical and technical contraints in relation to the environmental structure because their individual action capabilities might affect their visuomotor complexity during pre-planning and execution in on-sight climbing (i.e., the layout of the route, van Knobelsdorff et al., 2020).

Embodied planning explicitly considers these interactions between cognitive and motor planning in climbing. The embodied planning process is a continuous, bi-directional feedback loop. Through this feedback loop, perceptual and cognitive planning processes in the pre-planning phase constantly interact with motor-planning processes occurring during the execution of a plan (Musculus et al., 2021). Therefore, the embodied planning process is constantly influenced by bodily as well as sensorimotor experiences. This means that “what” I plan to do will also affect “how” I do it when executing a task (Raab, 2017). We argue that in planning a complex task such as completing a climbing route, both the “what” and “how” are intertwined (Boschker et al., 2002) as embodied cognition approaches suggest (Raab and Araújo, 2019). We note that pre-planning and execution can be partly experimentally separated – looking at a climbing wall without moving at the wall can be considered as “rather cognitive” or “more cognitive” pre-planning, whereas during climbing, both the “what” and “how” components of embodied planning are more tightly interrelated, with motor and cognitive planning aspects interacting constantly. Importantly, both the “how” and “what” components of the plan as well as the interaction are likely to be influenced by the climbing experience (Sanchez et al., 2019; Musculus et al., 2021). Therefore, the present study tests whether climbers with more experience look for potential routes on the climbing wall and pre-plan climbing routes differently.

### The present study

The present study aims to test embodied planning in climbing and to capture the combined visual and motor performance of climbers. This aim will be delivered by exploring whether climbing experience is related to pre-planning the route during route preview and to motor performance during route execution. We envision a better understanding of embodied planning processes and potential effects of experience by analyzing which holds climbers look at during route previewing and which holds they use when climbing the wall (Musculus et al., 2021).

We hypothesized that holds that were fixated on more often and for longer during the pre-planning of the route would be more frequently used when executing the plan in climbing the route. This effect should be accentuated for expert climbers compared to less experienced climbers.

## Materials and methods

### Participants

*A priori* power analysis performed with the G*Power 3.1.9.7 software (Faul et al., 2007) was used to calculate the required sample size for this study. The results showed that a minimum sample size of 15 participants were required (settings: paired sample t-test, *d* = 0.80, alpha level = 0.05, power = 0.8). To satisfy this sample requirement, we recruited 18 male climbers (*M*age = 26.56 years old, *SD* = 4.73). The sample followed previous climbing studies with gaze measures (*N* = 18 in Seifert et al., 2017; *N* = 12 in Nieuwenhuys et al., 2008). The climbing performance level of the participants’ *on-sight* style was between the 18 and 28 levels of the International Rock Climbing Research Association (IRCRA), reporting a scale of 6b + and 8b in the French scale (see Draper et al., 2011, 2015 for a comparison of different grading scales). We also measured their height (*M* = 170.53 cm, *SD* = 11.19), arm span (*M* = 176 cm, *SD* = 9.19), ape index (i.e., the measure of the ratio of an individual’s arm span relative to their height; *M* = 1.03, *SD* = 0.03), weight (*M* = 68.38 kg, *SD* = 7.38), maximum specific finger strength of the right hand (*M* = 370 N, *SD* = 74.20), and maximum specific finger strength of the left hand (*M* = 378.30 N, *SD* = 82.40). All participants had a license for climbing competitions. They had accumulated a mean climbing experience of 8.22 years (*SD* = 4.45) and had trained in climbing for 2.94 years (*SD* = 2.96). The frequency of training was 3–4 sessions per week, and each training session lasted at least 1 h. For simplification, we refer to all climbers as “experienced” and test different degrees of experience on pre-planning and climbing behavior in our hypotheses.

Participants voluntarily took part in the study and provided informed consent prior to the start of the experiment. The study was performed according to the ethical standards of research of the university in accordance with the Declaration of Helsinki (2013). Specifically, this study received approval from the Bioethics and Biosecurity Committee on 6 March 2018 (n° 33/2018). Participants received general information about the research contexts and signed an informed consent form, but were naïve to the specific hypotheses.

### Materials

#### Climbing test

In the present study, a tiltable climbing wall was built to reproduce representative situations of boulder climbing championships. This wall in the laboratory was engineered based on (i) the French grading system of difficulty, being most commonly used in mainland Europe, and (ii) competition regulations of the IFSC (2022) (e.g., the routes limiting the risk of falling that result in injuries, no downward jumps required, starting holds/top clearly marked with one color). The design of the climbing wall was supported by advice from five expert coaches of a Regional Mountain and Climbing Federation with more than 10 years of climbing experience. One coach was also an experienced setter in national official climbing competitions. They set the route following some of the boulder rules of the IFSC (2022) with a variety of sizes and shapes, leading to the creation of different climbing routes of medium–low difficulty levels (17 IRCRA level/6b + French scale). Therefore, all participants had the requisite minimum skill level to climb the wall.

**Figure 1:**
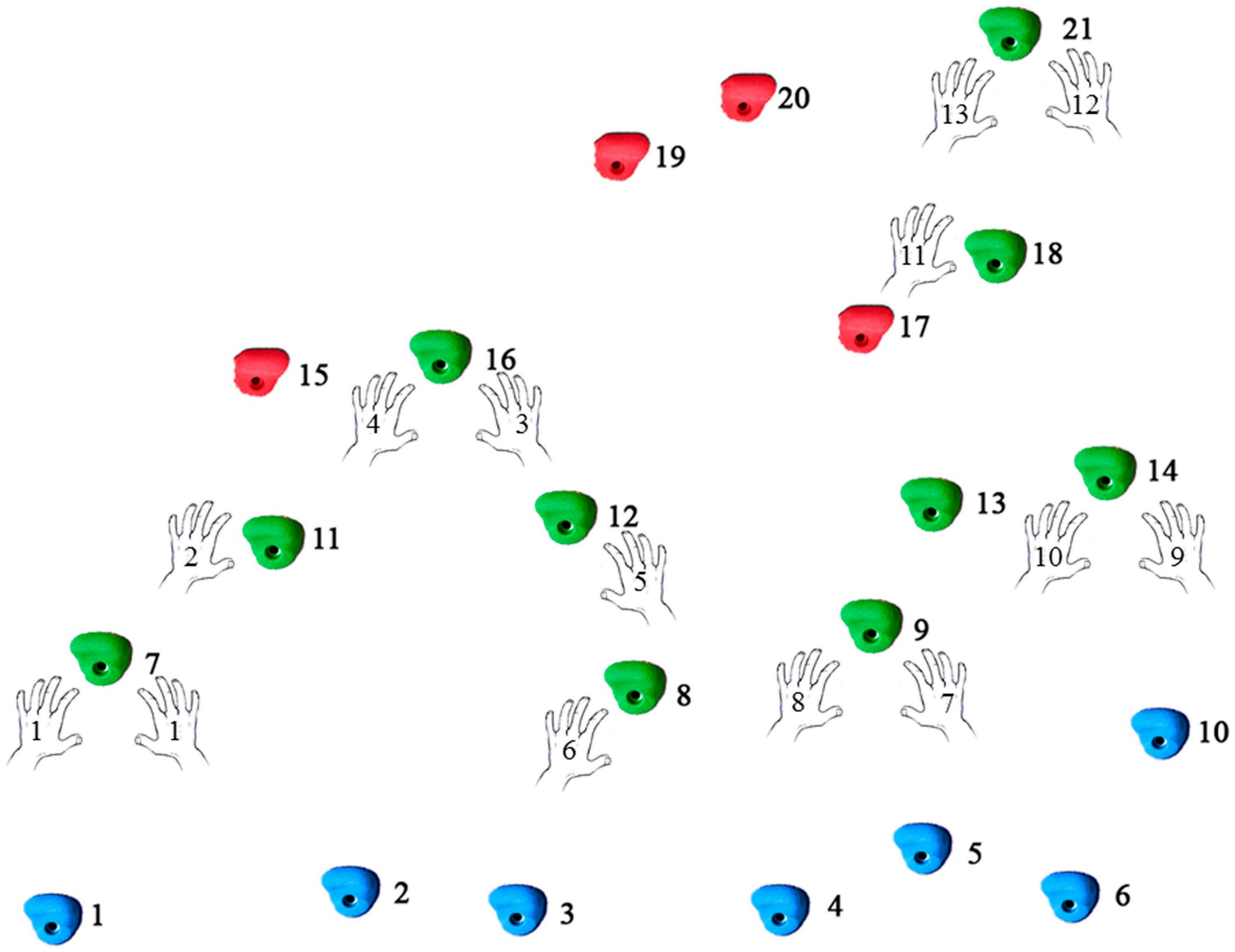
Locations of the hand-holds on the climbing wall (numbers 7-8-9-11-12-13-14-16-18-21 for the “key” hand-holds and 15-17-19-20 for deceptive hand-holds proposed by the expert coaches; number 1-2-3-4-5-6-10 for feet-holds).

The climbing wall contained a total of 21 foot- and hand-holds (footholds: from 1 to 6, and handholds: from 7–9 and 11–21). The best route for climbing was selected by a consensus of the expert coaches, containing a recommended sequence of 13 hand moves, using 10 holds (hand-holds numbers 7, 8, 9, 11, 12, 13, 14, 16, 18, and 21; see Figure 1). A number of alternative hand-holds were added to the wall to disguise which route would be the best for climbing it. For this purpose, some deceptive hand-holds offered a shorter but more complicated route (holds 15, 17, 19, and 20). The experts finally decided collectively the hand-holds needed to be gripped in a correct sequence to complete the best route to climb the wall safely. Altogether, we used a modified bouldering task with more handholds than 12 and the average number of 4–8 handholds per boulder as recommended by the IFSC (2022) to ensure the ability of climbers to visually inspect a boulder before ascent it.

We assessed individual factors that are known to affect climbing performance. First, a TANITA BC-418 bio-impedance scale was used for weight measurement, and a measuring tape was used for height and ape index parameters. Second, a SMEDLEY III hand dynamometer coupled to a wooden base was used for the measurement of the specific strength variable for climbers’ fingers. Each climber placed the forearm onto the base and fingers on the dynamometer in the form of a climbing grip (Morenas et al., 2013). Finally, a FANTEC BEASTVISION HD camera was used to record the measurement protocols and the later performance of the climbers on the wall.

### Pre-planning (during route previewing)

#### Visual search behavior

We used a mask-type eye tracker (Morenas et al., 2018) to capture gaze underlying expert performance (Gegenfurtner et al., 2011). Gaze parameters follow previous climbing studies (Nieuwenhuys et al., 2008; Grushko and Leonov, 2014; Seifert et al., 2017): the *number*, *duration*, and *sequence* of visual fixations that each climber performed on the holds of the climbing wall or beyond. In this study, a visual fixation was defined as the minimum time of 100 ms that the gaze remained stable at a location within 1.5° of the visual arc (Williams and Davids, 1998).

We measured the *visual performance* (*VP*) by comparing which of the hand-holds that experts had determined to be the ones to use to complete the route were actually looked at by the climbers. The holds determined by the experts are referred to as “key” holds. For example, if the fixations of climbers matched all hand-holds included on the route designed by expert coaches to climb the wall, then the climber achieved a 100% of *VP*. If they fixated at half of the holds, the value was 50% of *VP*. If they did not fixate their gaze at any of the designed holds, the value was recorded as 0% of *VP*. The climber had to perform at least one fixation on each of the 10 holds proposed by the coaches to achieve the 100% value. The designed route was not known by any of the participants.

### Motor execution (during route execution)

Climbers were asked to climb the route that was judged as most suitable to reach the top. We measured the *motor performance* (*MP*) by comparing which of the hand-holds that experts had determined to be the ones to use to complete the route were actually selected and used by the climbers during route execution. Equal to *VP*, *MP* was analyzed as a percentage. For example, if the climbers placed their hands on all hand-holds that were previously determined by the experts, they achieved a 100% for *MP*.

In addition, variables related to climbing performance used in past climbing studies (Nieuwenhuys et al., 2008; Sanchez et al., 2012) were included in the analyses. For example, the total climbing time (*Tclimb*) was the time that the climbers took to complete the climbing wall, being recorded from when they raised both feet off the ground until the moment that they placed both hands on the last hand-hold of the climbing sequence. We also recorded whether the climber completed the route on the wall (*Froute*) and the number of trials needed to complete a route successfully (*Ntrial*).

### Procedure

**Figure 2:**
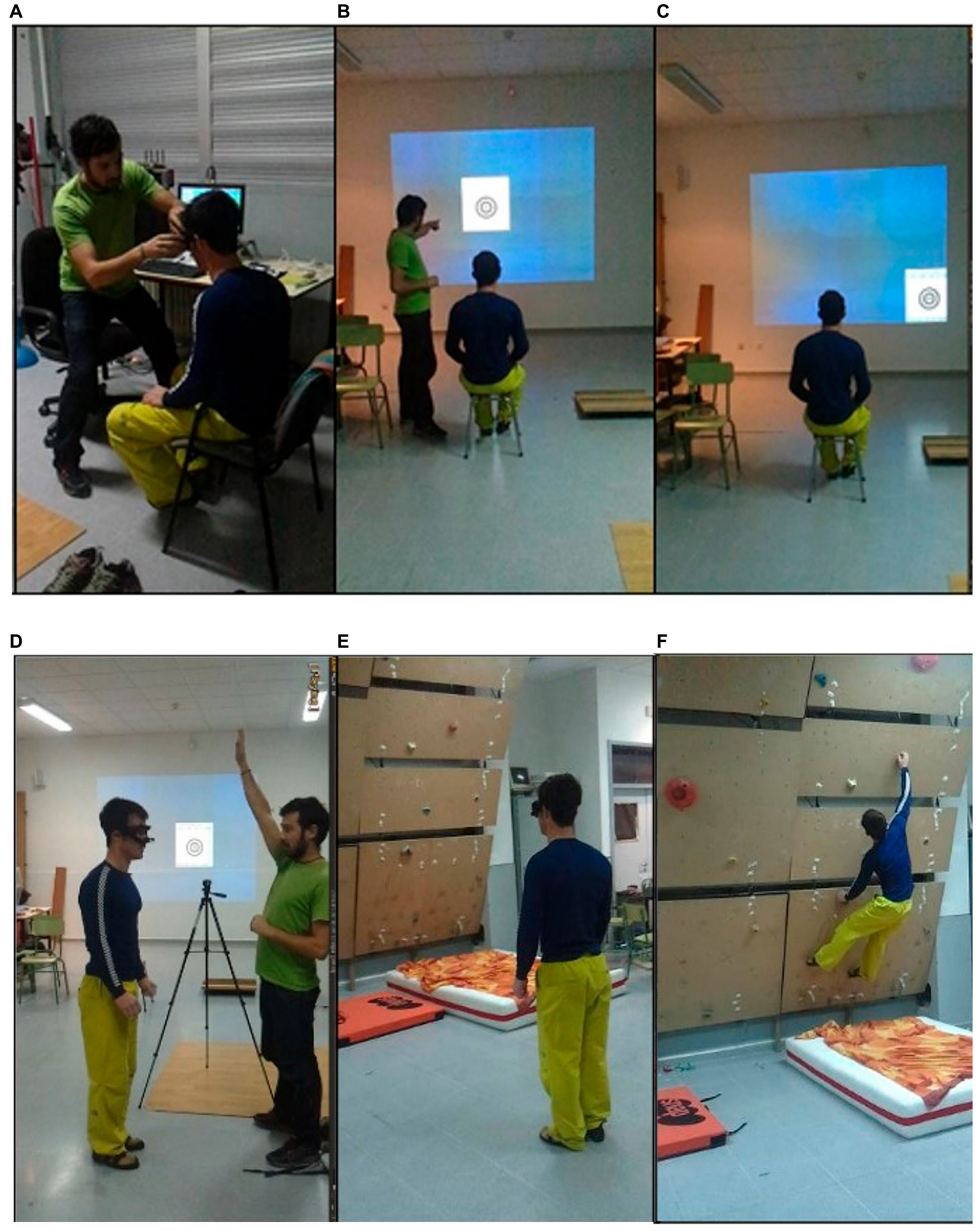
Procedure: Positioning the eye tracker **(A)**. Instructions about calibration process **(B)**. Calibration process **(C)**. Participant receiving instructions **(D)**. Visualization phase **(E)**. Performance phase **(F)**.

Each participant used his own climbing equipment. After a specific climbing warm-up near to the laboratory, each climber performed the anthropometric and specific finger strength measurements in a side-room. Then, the climber was directed to the only illuminated area of the room (calibration zone) where the eye-tracking device was attached to the participant’s head (Figure 2A).

The participant sat on a stool with a height-adjustable chin rest to look at the projection wall. The calibration protocol began (Figure 2B) with the projection of the nine calibration points, with climbers instructed to view the markers only with eye movements (Figure 2C). When this process finished, the participant stood up with his back to the climbing wall at a distance of 4 m in a delimited area of 1×1 m.

Once there, the lights were turned on, and the participant heard the final instructions: “You must look through the route in front of you as you do in competitions or training sessions. The route starts at the red hold marked on the lower left side and ends in a red hold marked on the upper right.” The climber had a maximum of 2 min to observe the boulder before trying to climb it. The researcher (Figure 2D) signaled to turn and look at the route from the delimited area (Figure 2E). After this, the participant was invited to complete the route to the top (Figure 2F). They had several trials to climb the wall although they strived to ascend it at the first attempt.

### Statistical analysis

**Figure 3:**
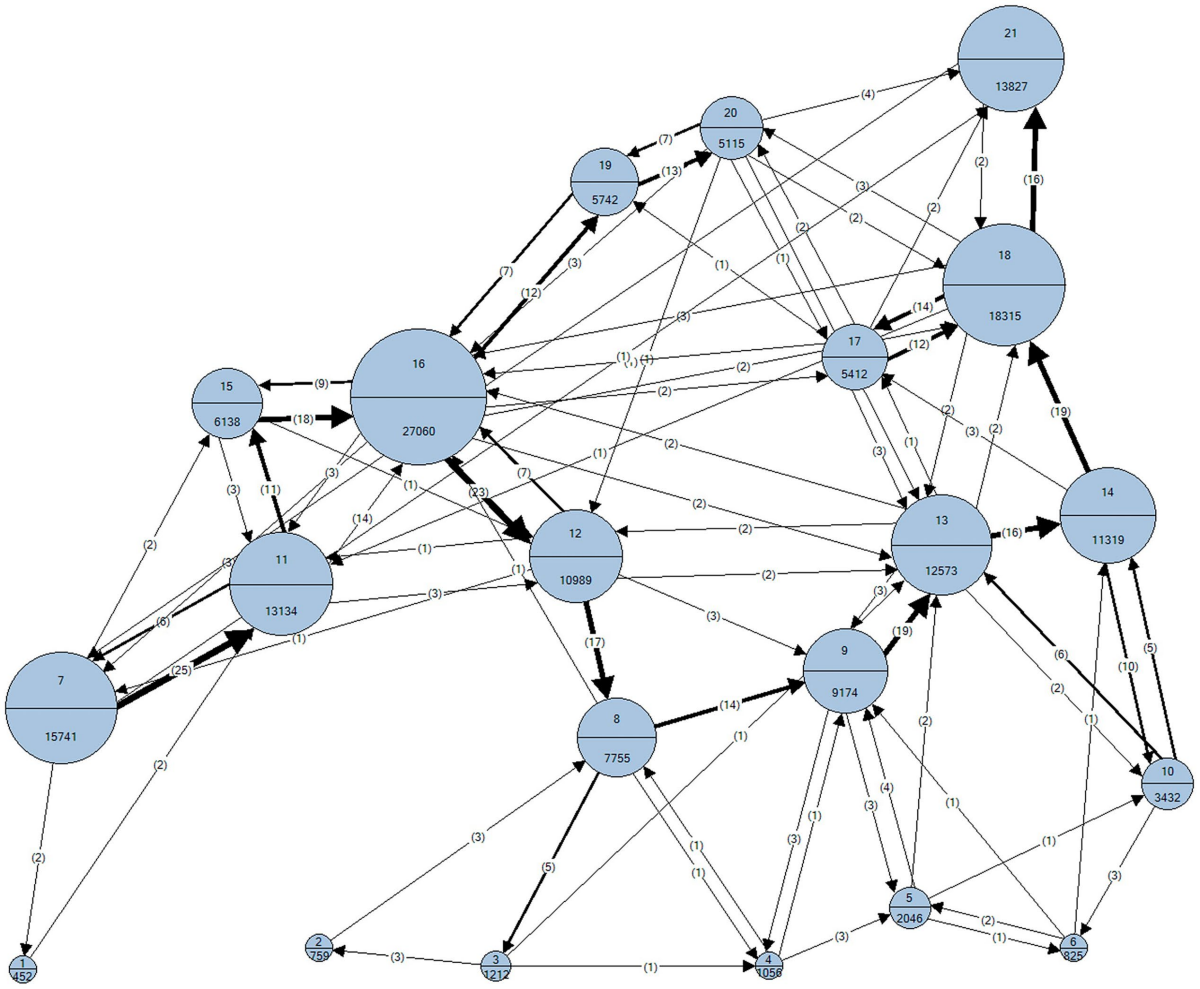
Gaze sequence of climbers during route previewing.

Nonparametric tests were used in this study because the Shapiro-Wilks analysis confirmed that the data of the dependent variables did not display a normal distribution. We performed descriptive statistics to analyze pre-planning regarding the sequence of holds looked at and used (see Figures 3 4), how often holds were looked at during route previewing, and how often they were used when executing the plan (i.e., route execution). We used the frequency distribution using Chi^2^ tests to analyze whether holds that were looked at vs. not looked at were more often used during the execution of the route.

**Figure 4:**
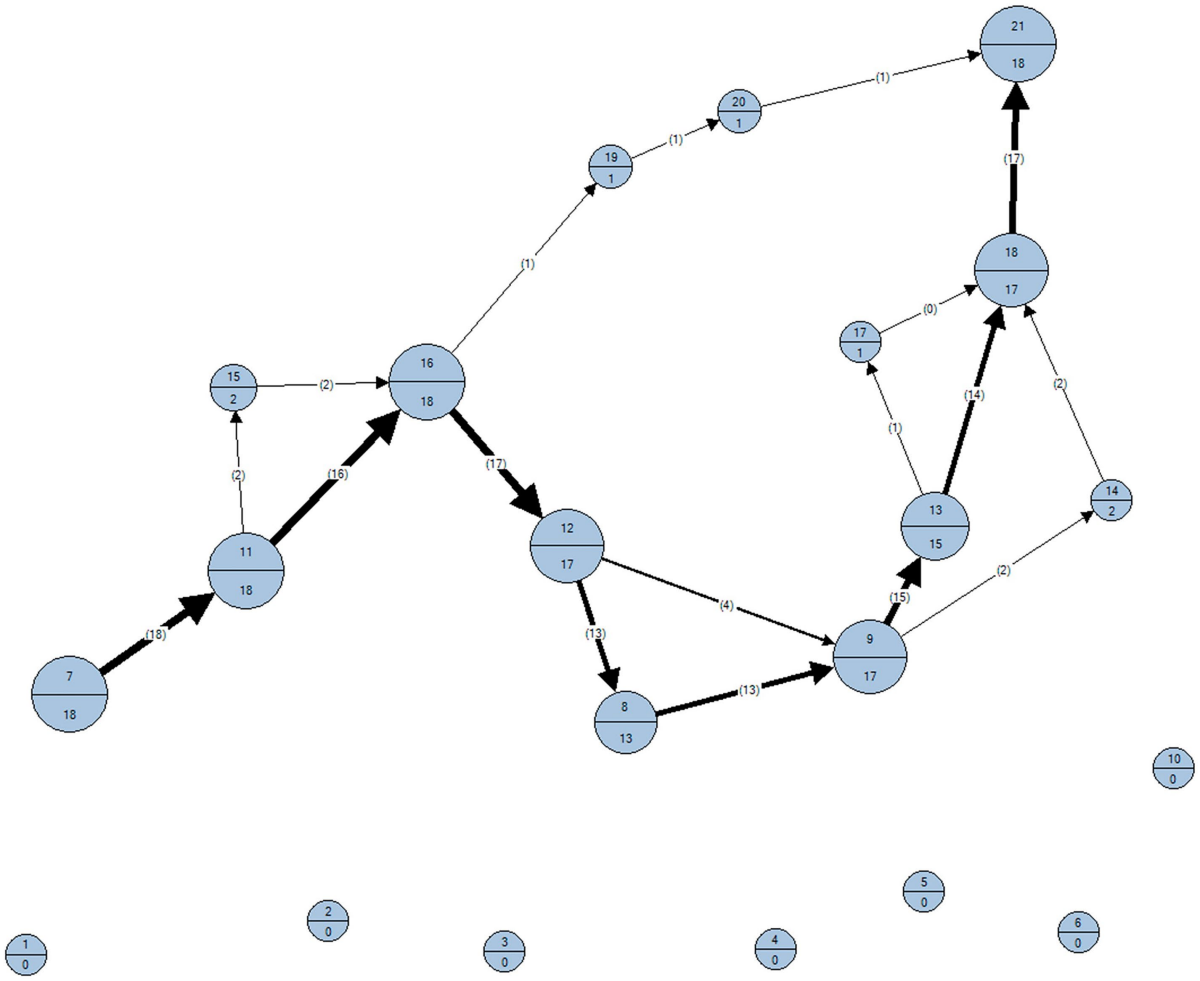
Grasping sequence of climbers during route execution.

We performed correlational analyses to explore relations between visual performance (*VP*), motor performance (*MP*), other variables related to visual search behavior (e.g., number and duration of fixations on the 21 holds of the climbing wall), and climbing performance (e.g., *Tclimb*, *Froute*, *Ntrial*) during route previewing and route execution. We also analyzed whether visual pre-planning and motor execution in climbing were related to experience (years of training). We did so by using correlational analyses and model fitting, in which we compared a linear, quadratic, and a growth function regarding model fit. Exploratorily, we computed and analyzed conditional gaze entropy following the procedure of Hacques et al. (2021). We did not see any significant relation to our study variables of interest. For interested readers, we added the descriptive statistics for each individual climber to the Supplementary material (please see Supplementary Tables 3 4 and 5).

An alpha level of <0.05 was set for all analyses. Statistical analyses were performed using the statistical package SPSS 21.0 (^©^ 2012 SPSS Inc.). The Grafos 1.3.5 software was used for the visualization of the sequence of fixations and hand-holds performance made by the climbers.

## Results

To better understand the interplay of pre-planning and route execution in climbing, visual search behavior and motor execution were analyzed. The sequence of visual fixations and motor execution performed by the climbers on the hand-holds of the climbing wall during route previewing and route execution are depicted in Figures 3 4, respectively. In these Figures, we observe that four of the 10 “key” hand-holds (holds 7, 11, 16, and 21) were looked at for longer times and used more often. For example, hold 16 was fixated at for approximately 27 s by the climbers and grasped 18 times during climbing execution.

### Effects of pre-planning on motor execution

Regarding the impact of pre-planning during route previewing on motor execution when completing the route, comparisons between the holds used during execution that were looked at before vs. not looked at before showed that significantly more holds were looked at than not looked at (*X*^2^ = 121.15, *p* < 0.001, φ = 0.86, *d* = 3.36). From this result, it can be inferred that during execution, the climbers relied on the holds they had visually scanned in their pre-planning during the route preview. Further, analyzing the holds looked at during pre-planning revealed that significantly more holds were later used than not used during route execution (*X*^2^ = 22.44, *p* < 0.001, φ = 0.09, *d* = 0.36) (see Table 1). This result suggests that looking at holds during pre-planning informs the subsequent use during route execution as further discussed below.

**Table 1:** Frequency distribution and percentage of holds looked at and not looked at during route pre-planning that were later used and not used during route execution.

		Gaze		
		Yes	No	Total
Used	Yes	159 (42.1%)	5 (1.3%)	164
	No	85 (22.5%)	129 (34.1%)	214
		244	134	378

We further analyzed whether the *duration* of fixations also differed for holds used vs. not used during route execution. The dependent *t*-test (*t*(17) = 5.10, *p* < 0.001, *d* = 1.20) revealed that, indeed, holds used were looked at longer (*M* = 754.56 ms; *SD* = 244.58; *SE* = 57.64) than holds that were not used during route execution (*M* = 396.80 ms; *SD* = 165.79; *SE* = 39.07). Together, the findings on the impact of route previewing on execution suggest that the visual search during pre-planning actually informs the motor performance during route execution as indicated in the Supplementary material (please see Video File S1).

### Relation between pre-planning and motor execution

Regarding the relation between pre-planning and motor execution of the routes execution, overall *VP* was significantly correlated with *MP* (*rho* = 0.796, *p* = 0.001). In addition, *Tclimb* and *Ntrial* were positively correlated (*rho* = 0.661, *p* = 0.003), which indicates that climbing performance was reliably measured in the experimental task.

Table 2 showed that *VP* and *MP* were negatively related to *Tclimb* and *Ntrial* in the climbing task. Correlational analyses also indicated that for three of the “key” holds (hand-holds 8, 14, and 18), the *VP* and *MP* of climbers were positively related to the fixation time, while the respective correlation was negative for the deceptive hand-hold 17.

**Table 2:** Significant relations (rho) between study variables.

	Visual performance	Motor performance
Climbing time	−0.892**	−0.887**
Number of trials	−0.546*	−0.572*
Fixation time on hold8	0.474*	0.740**
Fixation time on hold14	472*	0.595**
Fixation time on hold17	−0.634**	−0.719**
Fixation time on hold18	0.535*	0.578*

*p < 0.05; **p < 0.01.

This indicates that looking longer at some “key” holds was associated with better visual and motor performance, while looking longer at the deceptive hold was associated with worse visual and motor performance.

### The role of experience in pre-planning and motor execution

Regarding the role of experience in pre-planning the route during previewing and motor execution of the route, correlational analyses revealed that the number of years of climbing training was positively correlated to *VP* (*rho* = 0.560; *p* = 0.016) and *MP* (*rho* = 0.537; *p* = 0.021), and, as expected, negatively correlated to T*climb* (*rho* = −0.533; *p* = 0.023). There were no significant correlations of experience with the gaze strategies for holds looked at and used (*rho* = 0.414, *p* = 0.098), although experienced climbers with more years of training looked at holds not used for a significantly shorter amount of time (*rho* = −0.614, *p* = 0.009).

None of the models (linear, quadratic, growth function) yielded a significant fit for the duration of fixations for holds looked at and used. For the holds looked at but not used, the linear (*F*(1, 15) = 11.42, *p* = 0.004, *b*1 = −29.94, *R^2^* = 0.43), quadratic (*F*(2, 14) = 5.35, *p* = 0.019, *b*1 = −34.09, *b*2 = 0.44, *R^2^* = 0.43), and growth model (*F*(1, 15) = 16.22, *p* = 0.001, *b*1 = −0.082, *R^2^* = 0.52) were significant. The growth model with years of training explained 52% of variance.

## Discussion

Our study tested, in experienced climbers, how to pre-plan a climbing strategy during route previewing and how climbers actually perform the route during route execution. Our findings support the general notion of embodied planning that pre-planning and execution are intertwined (Musculus et al., 2021). This tight link between visual and motor performance in climbers is indicated by the correlations of hand-holds fixated at during pre-planning in the route preview and used during route execution. Further supporting the visual-motor link, visual performance was positively connected to motor performance. This can also be seen as an indicator of a reliable measure of expertise. Additionally, a positive correlation was found between the climbers’ visual performance and the duration of fixations on three prominent “key” holds pre-selected by the experts, and negative correlation with one deceptive hand-hold. Together, the findings of the present study demonstrate that the visual search during pre-planning actually informs motor performance during route execution. This suggests the dynamic interaction of visual and motor processes as suggested by embodied planning in climbing (Musculus et al., 2021).

Regarding the interpretation of results, we initially reasoned that the three “key” hand-holds of the climbing wall (holds 8, 14, and 18; see Figure 1) were positively associated with the visual and motor performance of the climbers because they constitute specific crux points (e.g., the most difficult parts of the route) constraining the perception of their action opportunities at the climbing wall (Seifert et al., 2017). This might favor a visual strategy that has previously been referred to as a “sequence of blocks,” consisting of scanning a route by chunking 2–4 hand-holds into a block. This strategy enables climbers to visualize alternative solutions while preparing for the route (Grushko and Leonov, 2014). According to Grushko and Leonov, this visual strategy is the most common visual pattern of climbers because it is connected to the tactical component of training. However, the climbers seemed to perform not only the “sequence of blocks” strategy but also an “ascending strategy” indicated by longer fixations at hand-holds 7, 11, 16, and 21 compared to other ones (see Figure 1). The “ascending strategy” is characterized by directing the gaze starting from below and then straight upwards, finishing the preview on the highest hold. Through this strategy, participants seem to inspect the most important (“key”) holds of the wall and their respective features (e.g., shape, orientation, size) in order to infer how reach, graps, or use them during route execution (Grushko and Leonov, 2014; Seifert et al., 2017).

More specifically, we firstly suggest that the time of route previewing provided climbers an opportunity to enrich their planning of perceptions (i.e., perceived events) and actions (i.e., to-be-produced events), leading to a decreased performance time in the climbing sequence. We reasoned that climbers exploited longer fixations on some “key” holds during the initial viewing of the route, based on their motor repertoire accumulated in climbing environments (i.e., action possibilities for long-term planning; see Rietveld and Kiverstein, 2014). Therefore, past climbing experiences could guide the gaze of climbers toward the relevant holds of the climbing wall during route previewing, even when they were not moving (i.e., an offline action specific effect on perception; Schütz-Bosbach and Prinz, 2007). This effect is relevant for an understanding of what is simulated during the pre-planning phase and adds to previous work that focused on climbing (e.g., Pezzulo et al., 2010; Bläsing et al., 2014).

Second, we argue that the holds more fixated on during route previewing were used during the execution of the route because the perception of these holds helped climbers to identify specific targets during “route reading” and to plan the forthcoming actions required to climb it, also before climbing the route (Seifert et al., 2017). From this view, the initial observation of climbing holds would evoke the corresponding reaching and/or grasping actions needed to effectively ascend the route (Vainio et al., 2008). Therefore, the initial observation of a climbing wall would activate a motor simulation of specific climbing movements, providing climbers with a prior estimate of their action possibilities to climb the wall (Pezzulo et al., 2010). Importantly, the present climbing study is – to the best of our knowledge – the first to use analyses establishing the direct relation between gaze (holds looked at; see Figure 3) and motor execution (holds used; see Figure 4). By analyzing the conditional frequencies and mean fixation times, this study demonstrates the functional connection of gaze behavior and motor execution.

Third, embodied planning strategies in climbing, such as how often and how long holds were looked at and used during route execution, were related to experience (years of training). Results revealed that, with more experience, climbers looked at holds not used for significantly shorter times and climbed faster. Indeed, a significant correlation was found between experience and fixation times on holds not used. Thus, the years of training were positively correlated to their visual and motor performance when climbing the wall. With these data, we argue that a refinement between visual information and movement patterns emerged in the experienced climbers of this study as a consequence of repeated expositions to specific routes and actions performed in climbing environments. It seems that more experienced climbers, in contrast to less experienced climbers, have learned which stimuli (here, hand-holds) are more informative than others for their overall goal of successfully ascending the climbing wall. This finding can be interpreted in the light of the “education of attention,” a frequently reported learning process resulting in the specification of informational stimuli and information pick-up (van der Kamp and Renshaw, 2015). As a result, these specific experiences would lead to the benefit of a better interpretation of the climbing-route information available prior to climbing the wall because they enable the experienced climbers to focus more on the respective function of the information (Boschker et al., 2002). Previously, Hacques et al. (2021) found that both exploratory hand movements and visual activity of intermediate climbers were used to gather information about different climbing routes. Specifically, these climbers displayed fewer fixations, decreased the number of fixated areas of interest, and performed less exploratory hand movements following a learning period on a climbing wall. The authors reasoned that these findings could reveal a reorganization of climbers’ gaze behavior with learning in a more structured order to better guide their forthcoming climbing actions.

Altogether, the visual behavior of climbers with more experience in this study seems to be more economical and focused on some hand-holds that are more informative than others (i.e., related to the overall goal of successfully ascending the climbing wall), compared to the visual patterns of climbers with less experience. Therefore, the expertise of climbers, gained after years of practice and training in indoor climbing, drove their gazes effectively to the relevant stimuli available in this specific environment. Additionally, expert rock climbers show not only a better cognitive processing of perceptual information but also a better visual “hardware” as indicated by a psychophysical optical tests (Marcen-Cinca et al., 2022).

These expertise differences between athletes of different skill levels regarding fixation location and fixation duration have been previously tested within the expert/non-expert paradigm (Mann et al., 2007; Gegenfurtner et al., 2011). For example, Gegenfurtner et al. (2011) found that expert athletes displayed shorter fixation durations, fixating more frequently at task-relevant areas and less on task-redundant areas, compared with non-experts. Similarly, Mann et al. (2007) concluded that expert performers´ eye movements were characterized by fewer fixations of longer duration than less skilled experts. Within the sport of climbing, a discriminating variable of the skill level of climbers has been the visual information they focus on. For example, Seifert et al. (2017) claimed that expert climbers used the time of route previewing more efficiently to link the visual information about reachable and graspable holds of a climbing wall with accurate climbing movements. These differences in visual search strategies have been also found in climbing coaches of different skill levels because experts fixated mostly at relevant aspects of a climber’s movement, using fewer fixations of greater duration, compared to the novice climbers (Mitchell et al., 2020). Recently, Medernach et al. (2024) argued that skilled climbers in indoor climbing had superior memory abilities to remember more climbing holds and movements driven by better mental visualization and enhanced visual attention to functional aspects of boulders.

Before concluding, we would like to acknowledge the potential limitations of this study. First, our sample size was not very large but is close to the average of 20.6 participants that we found from reviewing 60 studies on natural visual search behavior in sports (Kredel et al., 2017). In addition, other past climbing studies with gaze measures used the same number of climbers as our study (*N* = 18 in Seifert et al., 2017) or even fewer (*N* = 12 in Nieuwenhuys et al., 2008). Therefore, it would be interesting in future studies to test whether the significant differences found with this small sample of climbers would remain for a higher number of participants. In line with this, the use of larger samples of climbers with major homogeneity regarding their climbing experiences in future climbing research designs, both in years and frequency of training per week, would ensure the testing of more reliable correlations between (visual and motor) climbing performance, holds looked at/not looked at, and holds used/not used.

Second, it can be argued that bodily information may be an important factor for climbers to succeed in pre-planning a route. Our measures did not allow us to quantify the body movement during the pre-planning phase, and we did not specifically instruct participants to use or not use their bodies, so we believe participants engaged their routine behavior. In future studies that go beyond our current investigations of gaze strategies during the pre-planning phase, it maybe worthwhile to quantify bodily composition, capability, and movements of climbers, relating them to the climbing performance.

Third, we used different numbers of deceptive and target holds in our setting that may reflect the realistic and valid situation that multiple ways in planning a route are potentially good, resulting in more target holds and fewer deceptive holds. However, it could be argued that using 10 target holds and four deceptive holds might produce an effect that a climber who uses all target holds to reach the goal will potentially look at more target holds compared to deceptive holds. This effect could be systematically investigated in future studies using different relations of target and deceptive holds.

## Conclusion

The present study shows that the pre-planning processes captured through visual search behavior inform the motor execution when climbing a route. In addition, this study is the first to provide empirical evidence on the proposed link between perceptual-cognitive processes of an initial planning phase and motor processes when executing the plan (i.e., embodied planning in climbing; see Musculus et al., 2021). The findings presented here reinforce the contribution of previous specific motor experiences on action perception (Beilock, 2008), known as the “motor experience hypothesis” (Cañal-Bruland et al., 2010) and often discussed in the context of embodiment in sports (Raab, 2017). Specifically, the accumulation of climbing experiences enhanced climbers´ action-perception coupling, enabling them to find hand-holds for better route completion.

## Data availability statement

The raw data supporting the conclusions of this article will be made available by the authors, without undue reservation.

## Ethics statement

The studies involving humans were approved by the Bioethics and Biosecurity Committee of University of Extremadura (Spain) on 6 March 2018 (nº 33/2018). The studies were conducted in accordance with the local legislation and institutional requirements. The participants provided their written informed consent to participate in this study. Written informed consent was obtained from the individual(s) for the publication of any potentially identifiable images or data included in this article.

## Author contributions

VL-dC: Conceptualization, Formal analysis, Funding acquisition, Investigation, Methodology, Writing – original draft. JM: Conceptualization, Data curation, Investigation, Methodology, Writing – original draft. LM: Formal analysis, Methodology, Writing – review & editing. MR: Methodology, Supervision, Writing – review & editing.
