# Supplementary material for: Embodied planning in climbing: how pre-planning informs motor execution
Source: Front Psychol. 2024 Feb 19;15:1337878. doi: 10.3389/fpsyg.2024.1337878 (PMC10911288; doi:10.3389/fpsyg.2024.1337878)
Supplement: Supplementary file 1 [file Data_Sheet_1.pdf]

Title

Embodied planning in climbing: How pre-planning informs motor execution

Running title

Embodied plan in climbing

Author

-Vicente Luis del Campo (Luis-del Campo, V.: VLC)[1]

ORCID ID: https://orcid.org/0000-0003-3749-3294

Co-authors

-Jesús Morenas Martín (Morenas, J.: JMM) [2]

ORCID ID: https://orcid.org/0000-0002-0814-0636

-Lisa Musculus (Musculus, L.: LM)[3]

ORCID ID: https://orcid.org/0000-0003-1350-7903

-Markus Raab (Raab, M.: MR)[4]

ORCID ID: https://orcid.org/**0000-0001-6546-1666**

Emails: viluca@unex.es[1], jesusmorenas@unex.es[2], l.musculus-schoenenborn@dshs-koeln.de[3], raab@dshs-koeln.de[4]

Affiliation

[1,2]Laboratory of Motor Control and Learning. Faculty of Sport Sciences. University of Extremadura. Avda. de la Universidad, s/n, 10003, Cáceres (Cáceres). Spain.

[3,4]Deutsche Sporthochschule Köln, Psychologisches Institut. Am Sportpark Müngersdorf 6, Nawi-Medi 303, 50933 Köln.

Correspondence concerning this article should be addressed to Vicente Luis del Campo, Faculty of Sport Sciences. University of Extremadura. Avda. de la Universidad, s/n, 10003 Cáceres (Cáceres). Spain. Phone: (+34) 927257460 (Ext.57841). Fax: (+34) 927257461.

Table 3

Conditional gaze entropy (GTE) values of each climber obtained from the route previewing

Climber	Conditional entropy (GTE)
Climber1	8.01
Climber2	6.44
Climber 3	3.92
Climber 4	3.58
Climber 5	3.92
Climber 6	6.09
Climber 7	5.84
Climber 8	7.50
Climber 9	6.50
Climber 10	8.93
Climber 11	9.60
Climber 12	5.34
Climber 13	5.17
Climber 14	11.99
Climber 15	9.92
Climber 16	0.00
Climber 17	4.00
Climber 18	3.92

Table 4

Information on demographics and study outcomes for each individual climber

Climber	Experience (years of training)	Number of trials	Froute	Number of fixations	Duration of fixations	Visual performance	Climbing time	Motor performance
1	9	1	yes	24	11,187	92	14,21	100
2	7	1	yes	30	12,573	83	15,35	100
3	14	2	no	22	9,801	79	21,09	82
4	14	1	yes	20	10,197	79	15,24	100
5	18	1	yes	20	10,329	90	14,98	100
6	6	1	yes	25	12,309	90	14,47	100
7	7	1	yes	23	10890	81	15,05	95
8	4	1	yes	27	9306	87	16,69	80
9	5	1	yes	26	9207	76	18,05	82
10	3	2	no	34	10626	71	24,25	75
11	15	3	no	32	9867	73	24,72	52
12	7	3	no	35	9966	36	25,36	69
13	6	1	yes	22	8679	89	17,25	91,4
14	12	1	yes	33	9504	78	21,58	73
15	7	1	yes	31	9141	83	18,02	71
16	6	4	no	12	5676	66	26,14	56
17	5	1	yes	17	6303	40	23,85	51
18	3	1	yes	18	6435	50	25,4	54

Table 5

Fixation locations displayed by climbers at the holds of the climbing wall during route previewing

Climber	Number of visual fixation																																	
	1	2	3	4	5	6	7	8	9	10	11	12	13	14	15	16	17	18	19	20	21	22	23	24	25	26	27	28	29	30	31	32	33	34	35
1	7	1	11	7	15	16	12	16	7	11	16	12	8	9	13	14	18	10	6	5	13	14	18	21											
2	7	11	16	15	16	15	16	12	8	16	19	20	19	16	12	8	9	4	5	9	13	14	10	14	10	14	18	17	18	21					
3	7	11	15	16	15	16	12	8	3	2	8	9	13	14	18	17	13	14	10	14	18	21													
4	7	11	16	15	16	19	20	16	12	8	3	2	8	9	13	14	10	13	18	21															
5	7	11	16	15	16	12	8	3	4	9	13	14	18	17	13	14	10	13	18	21															
6	7	11	15	16	11	7	11	16	12	8	3	2	8	9	13	14	18	13	14	10	14	18	17	18	21										
7	7	1	11	7	15	16	12	16	15	16	12	8	9	13	14	18	13	9	5	13	14	18	21												
8	7	11	15	11	7	11	16	19	20	19	16	12	8	9	13	9	4	5	9	13	14	10	14	18	17	18	21								
9	7	21	7	11	15	16	11	16	12	16	19	20	19	16	12	8	9	4	5	10	13	14	18	17	18	21									
10	7	11	16	15	11	16	19	20	16	12	8	4	8	9	5	9	13	16	12	8	9	13	10	13	14	18	17	18	20	19	16	17	18	21	
11	7	11	7	11	16	12	16	19	20	21	18	17	16	7	11	12	8	9	13	16	13	14	10	13	17	18	16	19	20	17	18	21			
12	7	11	15	16	12	7	11	15	16	19	20	19	16	12	8	3	13	14	10	6	5	9	13	14	17	13	14	17	18	21	20	19	16	18	21
13	7	11	15	16	19	20	21	18	16	7	11	16	12	8	9	13	14	18	20	18	17	21													
14	7	11	16	12	11	15	12	16	19	20	19	16	11	7	11	15	16	17	20	13	12	8	9	13	10	6	14	18	17	18	16	18	21		
15	7	11	16	15	11	12	16	15	16	19	20	12	8	9	13	12	13	14	10	13	9	5	6	9	13	14	18	17	20	18	21				
16	7	11	16	12	9	13	14	18	17	19	20	21																							
17	7	11	15	16	13	14	18	17	21	7	11	12	13	14	17	18	21																		
18	7	11	15	16	12	16	19	20	16	12	9	13	14	18	17	18	20	21																	
